# Synergistic bacterial‒fungal interactions modulate the fermentation quality and in vitro degradation rate of triticale silage

**DOI:** 10.1186/s40104-026-01478-9

**Published:** 2026-08-03

**Authors:** Maoya Li, Yao Lei, Yulian Chen, Jiachuhan Wang, Yuanyuan Zhao, Xiangjiang He, Xiaoqing Zhang, Qiming Cheng, Chao Chen

**Affiliations:** 1 College of Animal Science, Guizhou University, Guiyang, 550025 China; 2 Grassland Research of Chinese Academy of Agricultural Sciences, Hohhot, 010013 China

**Keywords:** *Aspergillus niger*, Bacterial community, *Delftia*, In vitro degradation rate, *Lactiplantibacillus plantarum*, Metabolome, Triticale silage

## Abstract

**Background:**

The humid climate and frequent rainfall during the harvest season substantially hinder the utilization of triticale as feed. Although ensiling technology can effectively preserve nutrients, its fermentation quality depends on complex microbial interactions, the core mechanisms of which remain unclear. This study proposes and validates the hypothesis that “bacterial–fungal synergy” can enhance silage fermentation. By inoculating triticale silage with *Aspergillus niger* (AN), *Lactiplantibacillus plantarum* (LP) or their combination (ANLP) and performing multi-omics analyses, the mechanism underlying this synergistic effect was systematically elucidated in this study.

**Results:**

Compared with the control treatment, triticale silage inoculated with ANLP presented significant decreases in the neutral detergent fiber (NDF), acid detergent fiber (ADF), and ammonia nitrogen (NH_3_-N) contents and significant increases in the water-soluble carbohydrate (WSC), crude protein (CP), and lactic acid (LA) contents (*P* < 0.05). More crucially, ANLP treatment specifically enriched *Delftia*, indicating a special functional role for this bacterium in triticale silage. Further metabolomic and correlation analyses revealed that the synergy between *A. niger* and *L. plantarum* not only promoted the proliferation of *Delftia* but also activated the phenylalanine, tyrosine, and tryptophan biosynthesis pathways. This activation drove the synthesis of phenolic acid compounds with antimicrobial and antioxidant activities, such as coumaric acid and indole derivatives. These bioactive metabolites effectively inhibited the growth of harmful microorganisms. In vitro digestibility trials confirmed that the ANLP-treated group achieved the highest dry matter and protein degradation rates, thereby validating the pathway from the microbial mechanism to end-use feed value.

**Conclusions:**

Overall, the synergistic effects of bacteria (*L. plantarum*) and fungi (*A. niger*) can improve the fermentation quality and nutritional content of triticale by promoting amino acid metabolism and increasing the production of bioactive substances, providing a new strategy for increasing its utilization as a feed resource for ruminants.

**Supplementary Information:**

The online version contains supplementary material available at 10.1186/s40104-026-01478-9.

## Introduction

With the rapid growth of animal husbandry, shortages of high-quality feed have increased, necessitating the development of novel, high-quality feed sources. In recent years, triticale (*Triticum* × *Secale*), a novel crop synthesized via artificial chromosome doubling from wheat (*Triticum*) and rye (*Secale*), has been recognized as a highly promising high-quality forage crop due to its elevated protein content and robust stress resistance. This crop has been found to supply ruminants with ample protein and energy sources during the forage-scarce winter and spring seasons [1]. The humid climate in the southern regions of China, which is compounded by frequent rainfall during the triticale harvesting season, with precipitation generally reaching 300 to 500 mm per month, has significantly impeded the utilization of triticale as feed [1]. The application of ensiling technology has been identified as a viable solution to this challenge. Silage is produced via the anaerobic fermentation of fresh plant material, wherein lactic acid bacteria (LAB) convert water-soluble carbohydrates (WSC) to organic acids, creating an acidic environment that inhibits microbial proliferation and preserves the nutritional value of the forage [2]. However, the hollow stems of triticale retain substantial amounts of air, prolonging the establishment of an anaerobic environment during ensiling, which compromises fermentation efficiency and necessitates the application of additives to optimize the ensiling process [1]. The incorporation of microbial inoculants into silage has emerged as a prominent research focus in recent years and has been shown to have a variety of beneficial effects. Previous studies have demonstrated that the quality of sorghum silage can be improved through a reduction in the pH value and an increase in the acetic acid (AA) content when the silage is inoculated with *Lactiplantibacillus plantarum* [3]. In addition, inoculation with LAB improves the fermentation quality of alfalfa silage, as evidenced by a decrease in the pH value and ammonia nitrogen (NH_3_-N) content coupled with an increase in the α-tocopherol content [4]. LAB remain the predominant microbial inoculants used in silage production, but other microbial inoculants, such as *Aspergillus niger,* are being increasingly employed in silage research. Zhai et al. [5] revealed that the inoculation of sorghum silage with *A. niger* not only increased its crude protein (CP) content but also modulated the microbial community, leading to an increased abundance of LAB within the silage. *A. niger* was assigned the generally recognized as safe (GRAS) status by the U.S. Food and Drug Administration [6] and is approved as a feed additive in China; it also produces diverse enzymes (amylase, cellulase, pectinase, etc.) with activity across a pH range of 2.0 to 8.0, which is consistent with early-stage silage conditions [7]. Within this range, multiple enzymes can synergistically increase the nutritional quality of silage. To date, no studies have reported the effects of *A. niger* on the quality of triticale silage. Other studies have shown that silage inoculated with mixed microbial inoculants exhibits a superior fermentation quality to silage inoculated with individual microbial inoculants. For example, coinoculating triticale silage with *Bacillus coagulans* and *L. plantarum* increases the lactic acid (LA) concentration, aerobic stability, and relative abundance of LAB [1], whereas combined inoculation of *Silphium perfoliatum* L. silage with *L. plantarum* and *Levilactobacillus brevis* reduces the pH and NH_3_-N content and increases dry matter digestibility in vitro [8]. Despite these advances, the effects of the combined application of *A. niger* and *L. plantarum* on silage have not yet been reported.

The triticale silage fermentation process is driven by microbial enzymes that catalyze biochemical reactions, producing metabolites that dynamically interact with microbial communities to shape flavor and quality, with this mechanistic relationship now being systematically examined through metabolomics [9, 10]. Given the structural challenges of triticale (e.g., hollow stems delaying anaerobiosis), understanding the interplay between the metabolites and the microbial communities is essential for optimizing its ensiling process. Recent studies employing microbiome-metabolome integrated analyses have successfully elucidated the causal relationships between microbial functions and metabolite dynamics, providing a theoretical foundation for the targeted regulation of silage quality [10, 11]. However, the application of this dual-omics approach in triticale silage remains unexplored, hindering a deeper understanding of the intricate fermentation mechanisms involved.

Accordingly, the objective of this study was to examine the effects of single and combined inoculation with *A. niger* and *L. plantarum* on the fermentation attributes, bacterial microbiota, and metabolic profile of triticale silage through metabolomic and microbial community analyses.

## Materials and methods

### Materials and silage preparation

The primary material utilized in this experiment was triticale cultivated at the Guizhou Institute of Prataculture base in Dushan County, Guizhou Province, China, which is situated at a longitude of 107.54°, a latitude of 25.84°, and an altitude of 757 m. The average annual temperature is approximately 15 °C, and the average annual precipitation is approximately 3,000 mm. The harvested samples were cut into pieces ranging from 1 to 2 cm in length using a cutting machine. The inoculant strains were prepared as follows. *A. niger* (a strain sourced from Beina Chuanglian Biotechnology Research Institute, Beijing, China) was cultured on potato dextrose agar (PDA) at 28 °C for 7 d to induce sporulation. Spores were harvested by flooding the plate with sterile distilled water containing 0.05% (v/v) Tween 80, and the spore suspension was filtered through four layers of sterile cheesecloth. The spore concentration was determined using a hemocytometer and adjusted to 1 × 10^8^ colony‑forming units (CFU)/mL. Viability was confirmed by plate counting on PDA prior to inoculation. *L. plantarum* (a strain supplied by Zhongke Jiayi Biological Engineering Co., Ltd., Shandong, China) was revived from lyophilized stock in de Man, Rogosa and Sharpe (MRS) broth and incubated at 37 °C for 24 h under anaerobic conditions. The culture was centrifuged at 6,000 × *g* for 10 min at 4 °C, washed twice with sterile 0.85% NaCl, and resuspended in sterile distilled water to a final concentration of 1 × 10^8^ CFU/mL. Cell density and viability were verified by performing optical density measurements and MRS agar plate counting. The AN group was treated with *A. niger*; the LP group was treated with *L. plantarum*; the ANLP group was treated with a compound additive prepared by mixing equal volumes of *A. niger* and *L. plantarum* spore/cell suspensions to achieve a 1:1 ratio based on CFU; and the CK treatment group was treated with an equal volume of distilled water. An inoculation dose of 1× 10^6^ CFU/g fresh matter (FM) was selected based on previous studies demonstrating effective silage fermentation with similar inoculant levels [10, 12]. These treatments included six independent biological replicates, with each replicate containing 500 g of freshly chopped triticale (1–2 cm particle length). Triticale silage samples were packed into standard polyethylene bags (Deli Group Co., Ltd., Zhejiang, China; 30 cm × 40 cm, 0.12 mm thickness), vacuum-sealed to achieve a packing density of approximately 750 kg/m^3^ (corresponding to 95% compression), and stored in complete darkness at ambient temperature (21–30 °C) for 60 d. Both single- and mixed-strain bacterial suspensions were evenly sprayed onto the forage at a dose of 1 × 10^6^ CFU/g FM. The CP content of the fresh triticale was determined to be 12.45% dry matter (DM), the neutral detergent fiber (NDF) content was 58.35% DM, the acid detergent fiber (ADF) content was 34.32% DM, and the WSC content was 4.19% DM.

### Analysis of the chemical composition

The fresh triticale and triticale silage samples were placed in an oven at 65 °C for 72 h until a constant weight was achieved. The DM content was calculated using established methods described in previous research [11]. The dried samples were subsequently ground through a 1.0-mm sieve. The CP content was determined using the Kjeldahl method [13] with a Haineng K1100 automatic Kjeldahl nitrogen analyzer. The WSC content was measured using the phenol‒sulfuric acid method, as outlined by Owens et al. [14]. The NDF and ADF contents were assessed using the filter bag technique described by Van Soest et al. [15].

### Analysis of fermentation quality

The triticale silage samples (10 g) were blended with 90 mL of distilled water in a blender for 30 s to disrupt the cell walls, followed by filtration through four layers of medical gauze. The pH of the resulting filtrate was immediately measured using a pH meter. The concentrations of organic acids, namely, LA, AA, propionic acid (PA), and butyric acid (BA), were determined using an Agilent 1290 Infinity III LC system (Agilent Technologies, Santa Clara, CA, USA), employing the analytical methods and instrument settings described by Huang et al. [16]. The NH_3_-N concentration was measured using the method outlined by Broderick and Kang [17].

### Sequencing-based microbial analysis

Total microbial DNA was extracted from triticale samples through the CTAB method during fermentation, as detailed by Shuo et al. [18]. The purity and concentration of the DNA were determined via agarose gel electrophoresis, and the DNA was subsequently diluted to 1 ng/μL for use as a PCR template. For the analysis of the bacterial community, the full-length 16S rRNA gene was amplified with barcoded primers using Phusion High-Fidelity PCR Master Mix with GC Buffer. The amplification products were combined, separated on a 2% agarose gel, and recovered with a Qiagen kit. After purification, the library was constructed, quantified with a Qubit fluorometer and an Agilent 2100 Bioanalyzer, and sequenced on the Illumina MiSeq platform. For the analysis of the fungal community, the internal transcribed spacer 1 (ITS1) region was amplified using the primer pair ITS1F (5′-CTTGGTCATTTAGAGGAAGTAA-3′) and ITS2 (5′-GCTGCGTTCTTCATCGATGC-3′) with barcoded adapters. PCR amplification was conducted using Phusion High-Fidelity PCR Master Mix under the following conditions: initial denaturation at 98 °C for 1 min; 30 cycles of 98 °C for 10 s, annealing at 55 °C for 30 s, and extension at 72 °C for 30 s; followed by a final extension at 72 °C for 5 min. The PCR products were mixed, purified, and used to construct sequencing libraries using the TruSeq DNA PCR-Free Sample Preparation Kit. Library quality was assessed with a Qubit fluorometer and an Agilent 2100 Bioanalyzer, and paired-end sequencing (2 × 250 bp) was performed on the Illumina NovaSeq platform. Data analysis, encompassing principal coordinate analysis (PCoA), alpha diversity analysis, and redundancy analysis (RDA), was executed using the NovoMagic platform. For PcoA, the Bray‒Curtis dissimilarity metric was applied to the relative abundance data without additional data transformation or scaling. For the RDA, the species abundance data were Hellinger-transformed to reduce the influence of rare taxa, and the environmental variables were centered and scaled to unit variance. Sequences with ≥ 97% similarity were grouped into operational taxonomic units (OTUs) using UPARSE, and alpha diversity indices were calculated with QIIME software (version 2.15.3). Raw bacterial sequencing data have been deposited in the NCBI database under accession number PRJNA1328668. Raw ITS sequencing data have been deposited in the NCBI Sequence Read Archive under accession number PRJNA1457705.

*Delftia* were enumerated using a selective dilution plating method. Briefly, 10 g of triticale silage was homogenized with 90 mL of a sterile 0.85% NaCl solution and serially diluted. A 100 μL aliquot of each dilution was spread onto inorganic salt agar plates supplemented with 100 mg/L aniline as the sole carbon and nitrogen source. After an incubation at 28 °C for 48 to 72 h, colonies displaying the typical morphology were counted and the results are reported as log_10_ CFU/g FM.

### Metabolomic analysis

The changes in the metabolite levels of the triticale silage samples were investigated through an untargeted metabolomic analysis, which was conducted with the assistance of Beijing Novogene Co., Ltd. A 100 mg sample of triticale that had been pulverized in liquid nitrogen was mixed with 500 μL of 80% aqueous methanol in an Eppendorf tube, vortexed, and incubated on ice for 5 min. After centrifugation at 15,000 × *g* for 20 min at 4 °C, the supernatant was diluted to 53% with methanol, centrifuged again, and then analyzed using an Agilent 1290 Infinity III LC system coupled to an Agilent 6475 triple‑quadrupole mass spectrometer, with a Hypersil Gold C18 column and a mobile phase consisting of 0.1% formic acid in water and methanol. The data were processed with CD 3.3 software for metabolite screening, peak area correction with a QC sample, and quantification. The quality control (QC) sample was prepared by pooling equal volumes (10 μL each) of all individual sample extracts obtained after the initial centrifugation step. This pooled mixture was then subjected to the same dilution, centrifugation, and LC‒MS analysis procedures as the actual samples. The QC sample was injected periodically throughout the analytical run (every 10 injections) to monitor system stability and to allow correction of peak areas for batch effects. Partial least squares discriminant analysis (PLS-DA) was performed to identify metabolite features that discriminated between treatment groups. A sevenfold cross-validation method was applied to evaluate the predictive performance of the model, and the significance of the model was assessed using 200 permutation tests. Molecular formulas were predicted and matched against the mzCloud, mzVault, and Masslist databases. The normalized data allowed the identification of metabolites and determination of their relative quantities; the metabolites were annotated using the KEGG (https://www.genome.jp/kegg/pathway.html), HMDB (https://hmdb.ca/metabolites), and LIPID MAPS (http://www.lipidmaps.org/) databases. Our metabolomic data were uploaded to the NGDC OMIX repository, with the project number PRJCA046474.

### Determination of the in vitro degradation rate

The in vitro dry matter degradation rate (IVDMD), in vitro neutral detergent fiber degradation rate (IVNDFD), and in vitro crude protein degradation rate (IVCPD) after 48 h of incubation were determined using an in vitro simulated incubator, based on the methods described by Jin et al. [8]. Four Simmental cattle with an average body weight of 550 ± 30 kg in good growth conditions and fitted with permanent rumen cannulas were selected as donors for rumen fluid collection. The donor animals were fed a total mixed ration consisting of corn silage (40%), alfalfa hay (25%), and concentrate mixture (35%) twice daily at 07:00 and 19:00, with ad libitum access to fresh water and mineral blocks. The feed offered at 19:00 was typically completely consumed within 1 h, after which no further feed was provided, initiating a fasting period. Rumen fluid was then collected before the morning feeding at 07:00. The animal handling and rumen cannulation procedures were approved by the Institutional Animal Care and Use Committee of Guizhou University (Approval No. EAE-GZU-2026-E022), and all procedures were conducted in accordance with the university’s guidelines for the ethical use of experimental animals. Rumen fluid was collected from multiple sites within the rumen, pooled, and promptly transported to the laboratory in pre-warmed, CO_2_-flushed thermos flasks. The contents were thoroughly mixed, filtered through four layers of cheesecloth, and continuously flushed with carbon dioxide to maintain an anaerobic environment. The buffer solution consisted of 1.33 L of buffer A (10.0 g/L KH_2_PO_4_, 0.5 g/L MgSO_4_·H_2_O, 0.5 g/L NaCl, 0.1 g/L CaCl_2_·2H_2_O, and 0.5 g/L urea) and 266 mL of buffer B (15.0 g/L Na_2_CO_3_ and 1.0 g/L Na_2_S·7H_2_O). The buffer mixture was maintained at 39 °C under continuous CO_2_ purging. The bags were placed in glass incubation vessels containing 2 L of the pre-warmed buffer–rumen fluid mixture (1:2, v/v) and incubated at 39 °C for 48 h with gentle shaking (50 r/min). The residues were subsequently collected and washed under running tap water until the rinse water remained clear and then dried at 65 °C for 48 h to calculate the IVDMD, IVNDFD, and IVCPD [19]. The degradation rates were calculated using the following equations: IVDMD (%) = [(W_0_ − W_r_)/W_0_] × 100, where W_0_ is the initial DM weight of the sample and W_r_ is the DM weight of the residue after 48 h of incubation; IVNDFD (%) = [(NDF_0_ − NDF_r_)/NDF_0_] × 100, where NDF_0_ is the initial NDF weight of the sample and NDF_r_ is the NDF weight of the residue; and IVCPD (%) = [(CP_0_ − CP_r_)/CP_0_] × 100, where CP_0_ is the initial CP weight of the sample and CP_r_ is the CP weight of the residue.

### Statistical analyses

The data are reported as the mean ± standard deviation. Statistical analyses were conducted using IBM SPSS Statistics (version R26). One-way analysis of variance (ANOVA) was used to determine significance at *P* < 0.05, followed by Duncan’s multiple range test for post-hoc comparisons. The data were visualized utilizing GraphPad Prism (version 8.0; La Jolla, CA, USA) and R software (version 3.6.3).

## Results

### Chemical composition and fermentation quality of triticale silage samples after 60 days of ensiling

**Fig. 1:**
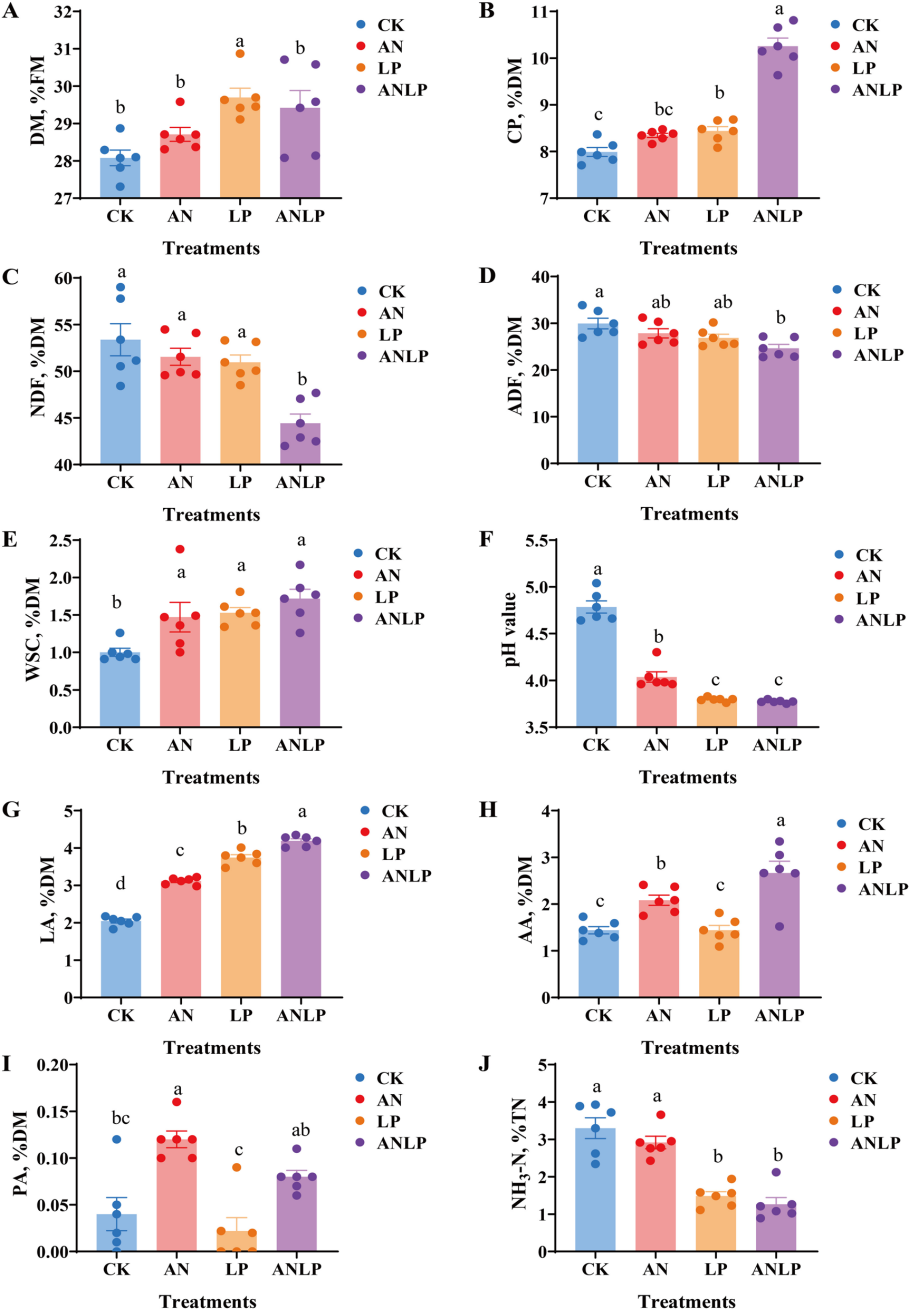
Chemical composition and fermentation quality of triticale silage samples after 60 days of ensiling. **A** Dry matter (DM, %FM). **B** Crude protein (CP, %DM). **C** Neutral detergent fiber (NDF, %DM). **D** Acid detergent fiber (ADF, %DM). **E** Water-soluble carbohydrate (WSC, %DM). **F** pH value. **G** Lactic acid (LA, %DM). **H** Acetic acid (AA, %DM). **I** Propionic acid (PA, %DM). **J** Ammonia nitrogen (NH_3_-N, %TN). CK, control group (no exogenous inoculant); AN, inoculation with *Aspergillus niger*; LP, inoculation with *Lactiplantibacillus plantarum*; ANLP, inoculation with *A. niger* and *L. plantarum*; TN, total nitrogen. The dots within each bar represent individual replicate values. Different letters indicate significant differences between treatment groups (*P* < 0.05)

The chemical compositions (DM, CP, NDF, ADF, and WSC) of the triticale silage samples after 60 days of ensiling are shown in Fig. 1A–E. Compared with those of the other groups, the DM content of the LP treatment group was significantly higher (*P* < 0.05; Fig. 1A). The CP content (Fig. 1B) in the CK treatment group was significantly lower (*P* < 0.05) than that in the other treatment groups, with the ANLP treatment group exhibiting the highest CP content. Compared with those in the CK treatment group, the NDF (Fig. 1C) and ADF (Fig. 1D) contents (*P* < 0.05) in the ANLP treatment group were significantly lower, whereas the WSC content (Fig. 1E) in the CK treatment group was significantly lower (*P* < 0.05) than in other treatment groups. The ANLP treatment group displayed the lowest NDF and ADF levels and the highest WSC content. The fermentation quality (pH value and LA, AA, PA and NH_3_-N contents) of the triticale silage sample after 60 days of ensiling is shown in Fig. 1F–J. The pH value (Fig. 1F) of the additive-treated groups (AN, LP, and ANLP treatment groups) was significantly lower than that of the CK treatment group (*P* < 0.05), with a concurrent increase in the LA content (Fig. 1G). Among the additive treatment groups, the ANLP treatment group presented the highest LA concentration. The AA content (Fig. 1H) was significantly higher in the ANLP and AN treatment groups than in the CK and LP treatment groups (*P* < 0.05). The PA content (Fig. 1I) in the AN treatment group was significantly higher than that in the CK and LP treatment groups (*P* < 0.05). The NH_3_-N contents (Fig. 1J) in the LP and ANLP treatment groups were significantly lower than those in the CK and AN treatment groups, with the ANLP treatment group exhibiting the most pronounced reduction (*P* < 0.05).

### Microbial community of triticale silage samples after 60 days of ensiling

**Fig. 2:**
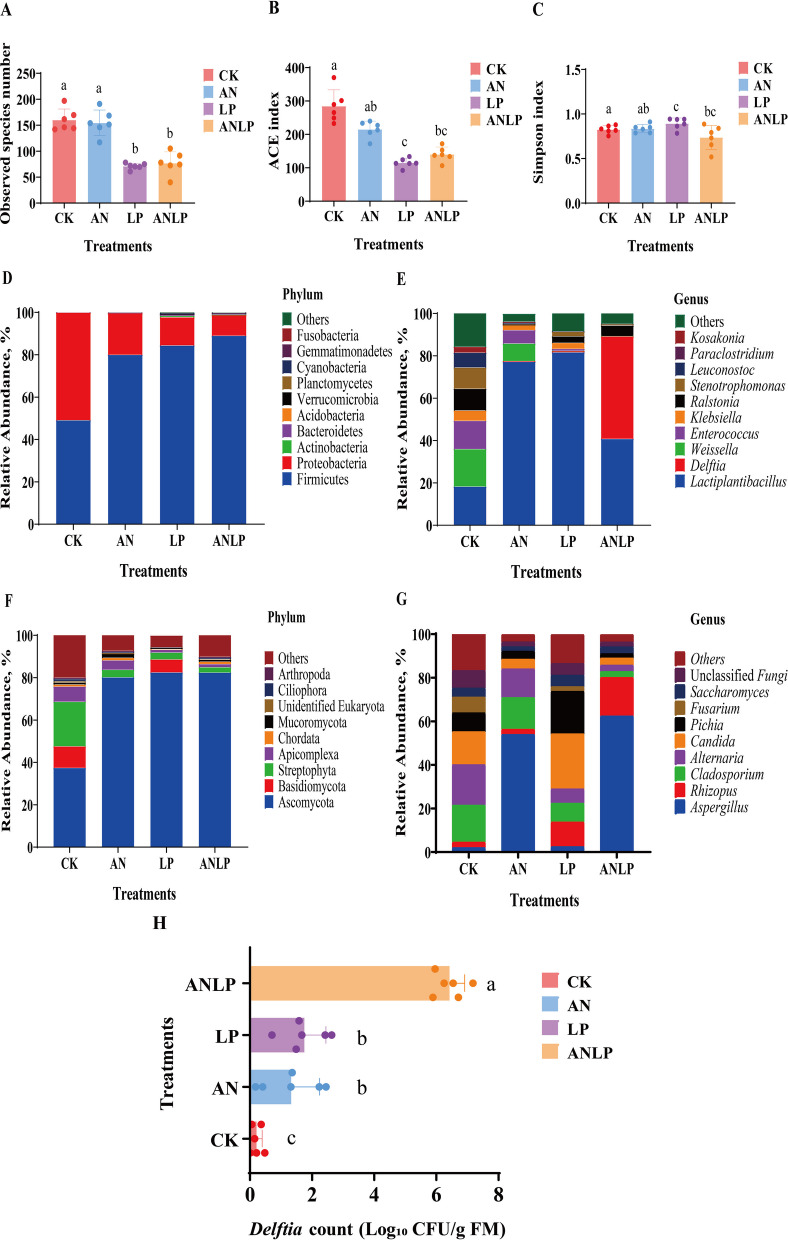
Microbial community of triticale silage samples after 60 days of ensiling. Alpha diversity indices: **A** observed species, **B** ACE index, and **C** Simpson index. **D** and **E** Relative bacterial community composition in triticale silage at the phylum (**D**) and genus (**E**) levels. **F** and **G** Relative fungal community composition in triticale silage at the phylum (**F**) and genus (**G**) levels. **H** Enumeration of *Delftia* in triticale silage after 60 days of ensiling. CK, control group (no exogenous inoculant); AN, inoculation with *Aspergillus niger*; LP, inoculation with *Lactiplantibacillus plantarum*; ANLP, inoculation with *A. niger* and *L. plantarum*. The dots within each bar represent individual replicate values. Different letters indicate significant differences between treatment groups (*P* < 0.05)

**Fig. 3:**
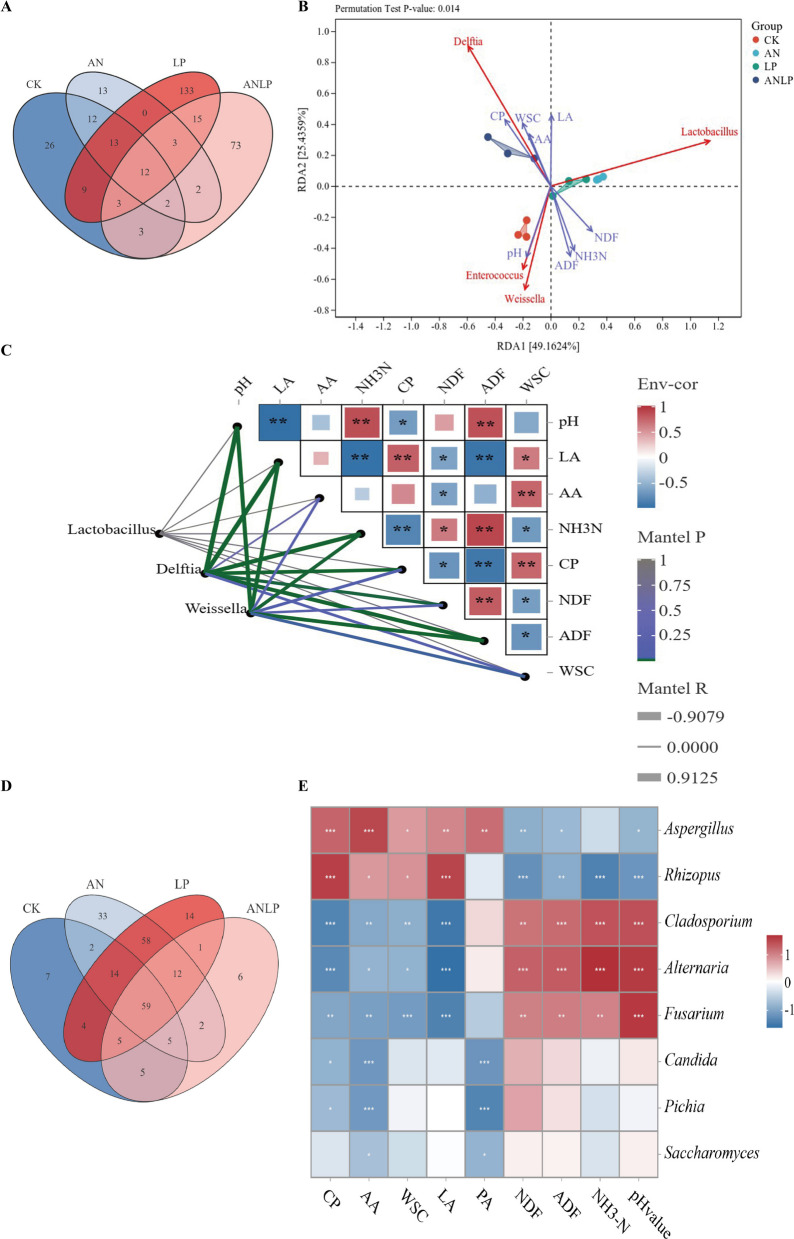
Microbial community composition and environmental factor correlations of triticale silage samples after 60 days of ensiling. **A** Venn diagram revealing the unique and shared bacterial OTUs of triticale silage. Correlations between the bacterial community and environmental factors were revealed by performing a (**B**) redundancy analysis (RDA) and (**C**) the Mantel test (the heatmap shows Mantel-based correlations between distance matrices, not standard pairwise correlations). **D** Venn diagram revealing the unique and shared fungal OTUs of triticale silage. **E** Correlations between the fungal community and environmental factors based on the Spearman correlation coefficient (*P* < 0.05). ^*^P < 0.05, ^**^P < 0.01, and ^***^P < 0.001. CK, control group (no exogenous inoculant); AN, inoculation with *Aspergillus niger*; LP, inoculation with *Lactiplantibacillus plantarum*; ANLP, inoculation with *A. niger* and *L. plantarum*. The dots within each bar represent individual replicate values

The alpha diversity indices (OTUs, ACE index, and Simpson index) of the bacterial community in triticale silage are shown in Fig. 2A–C. The ACE index and Simpson index were significantly lower in the LP and ANLP treatment groups than in the CK group (*P* < 0.05), indicating lower microbial abundance and diversity in the LP and ANLP treatment groups. Figure 2D shows the bacterial community composition in triticale silage at the phylum level, with Proteobacteria and Firmicutes identified as the predominant phyla. In the additive-treated groups, the relative abundance of Firmicutes increased and that of Proteobacteria decreased compared with those in the CK group. The bacterial composition at the genus level was analyzed in detail (Fig. 2E). In the CK treatment group, *Lactiplantibacillus*, *Weissella*, and *Enterococcus* were the predominant genera. In both the AN and LP treatment groups, *Lactiplantibacillus* was the dominant genus, although the AN treatment group additionally presented relatively high abundances of *Weissella* and *Enterococcus*. Notably, the ANLP treatment significantly increased the relative abundance of *Delftia*; prior to this study, reports on *Delftia* in silage were scarce, indicating a special functional role for this bacterium in triticale silage. The relative abundance of *Lactiplantibacillus* in the AN treatment group was higher than that in the CK treatment group, whereas the relative abundances of *Weissella* and *Enterococcus* were lower. Compared with the AN and LP treatments, the ANLP treatment resulted in a lower relative abundance of *Lactiplantibacillus* but resulted in superior fermentation quality. Figure 2F presents the phylum‑level composition of the fungal community in triticale silage, in which Ascomycota dominated. The relative abundance of Ascomycota increased in the additive‑treated groups compared with the CK treatment group. The genus‑level composition was analyzed in detail (Fig. 2G). In the CK treatment group, *Cladosporium* and *Alternaria* were the predominant genera. In the AN and ANLP treatment groups, *Aspergillus* was the dominant genus, whereas in the LP treatment group, *Candida* and *Pichia* were the dominant genera. The relative abundance of *Aspergillus* in the ANLP treatment group was higher than that in the AN and LP treatment groups. The relative abundances of *Cladosporium* and *Alternaria* in the ANLP treatment group were lower than those in the AN treatment group, whereas the relative abundances of *Candida* and *Pichia* in the ANLP treatment group were lower than those in the LP treatment group. As shown in Fig. 2H, the *Delftia* counts differed significantly among the four groups of treated triticale silage after 60 days of ensiling, and the number in the ANLP treatment group was significantly greater than that in the other treatment groups (*P* < 0.05). The beta diversity analysis revealed the influence of additives on both the bacterial and fungal compositions of triticale silage. Venn diagrams were generated to visualize shared and unique OTU counts within the bacterial (Fig. 3A) and fungal (Fig. 3D) communities. For bacteria, the unique OTU counts in the CK, AN, LP, and ANLP treatment groups were 26, 13, 133, and 73, respectively, with 12 OTUs common to all the treatment groups. For fungi, the unique OTU counts were 7, 33, 14, and 6, respectively, with 59 common OTUs. These results indicate that additives induced alterations in both bacterial and fungal community structures. RDA was employed to ascertain the specific factors driving the changes in the bacterial community, and the results revealed that RDA 1 and RDA 2 accounted for 49.16% and 25.44% of the variance, respectively (Fig. 3B), indicating that all the environmental factors and the four treatments differed significantly. The nutritional quality and fermentation attributes were strongly correlated with the bacterial community structure, as depicted in Fig. 3C. An in-depth interaction network analysis revealed that the abundance of the genus *Delftia* was significantly positively correlated with the LA (*R*^2^ = 0.913), CP (*R*^2^ = 0.752), and WSC (*R*^2^ = 0.509) contents but strongly negatively correlated with the pH value and the NH_3_-N (*R*^2^ = −0.905), NDF (*R*^2^ = −0.658), and ADF (*R*^2^ = −0.856) contents, as illustrated in Fig. 3C. Spearman’s correlation analysis was performed to investigate the associations between the fungal genera and the chemical composition and fermentation quality (Fig. 3E). *Aspergillus* was significantly negatively correlated with the NDF content, ADF content, and pH and positively correlated with the CP, AA, WSC, LA, and PA contents. *Rhizopus* was negatively correlated with the NDF content, ADF content, NH_3_-N content, and pH and positively correlated with the CP, AA, WSC, and LA contents. Conversely, *Cladosporium*, *Alternaria*, and *Fusarium* showed the opposite pattern and were negatively correlated with the CP, AA, WSC, and LA contents but positively correlated with the NDF content, ADF content, NH_3_-N content, and pH value. *Candida* and *Pichia* were negatively correlated with the CP, AA, and PA contents, whereas *Saccharomyces* was negatively correlated with the AA and PA contents.

### Metabolomic profiles of triticale silage samples after 60 days of ensiling

**Fig. 4:**
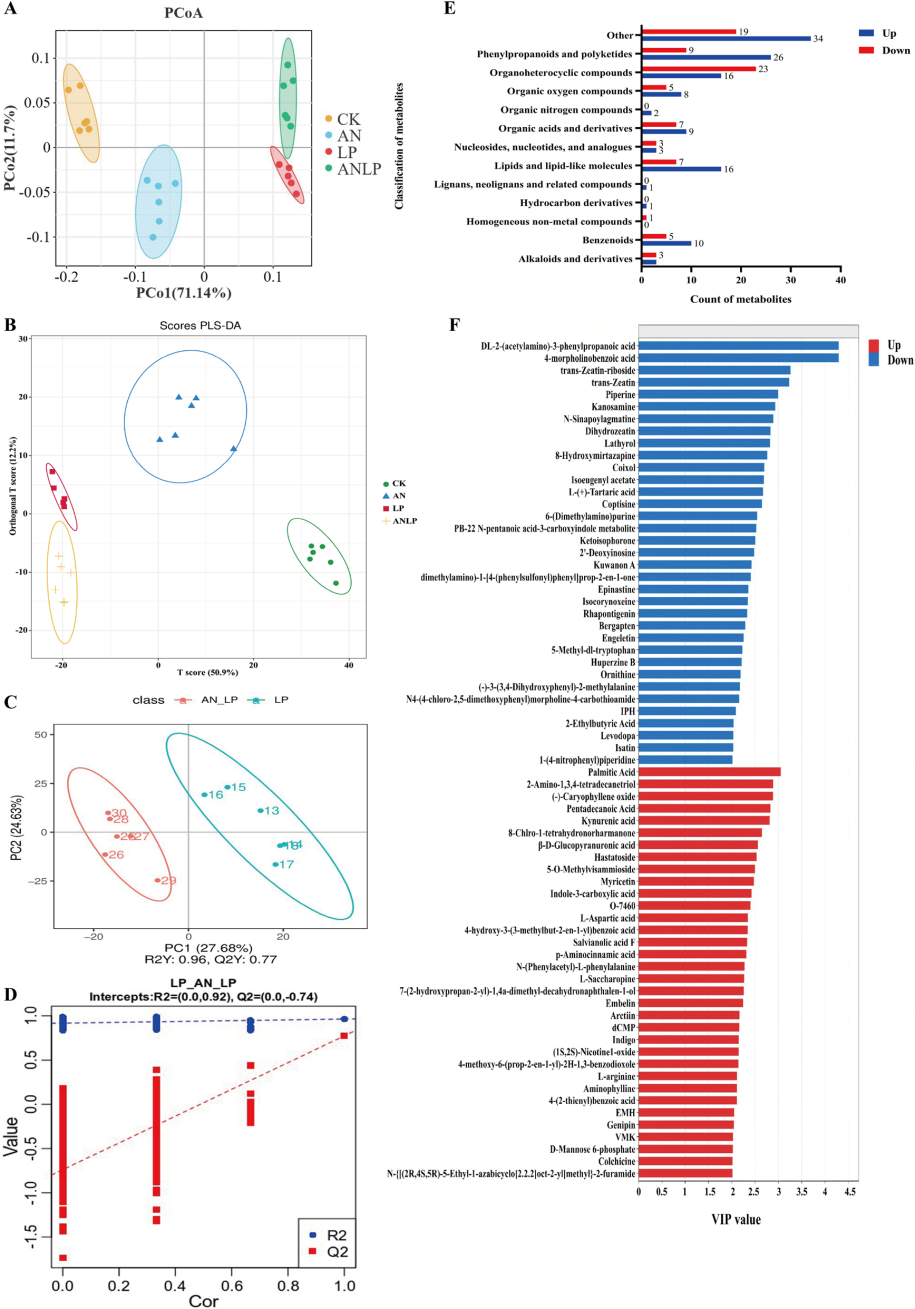
Metabolomic profiles of triticale silage samples after 60 days of ensiling. **A** Principal coordinate analysis (PCoA) score plot. **B** and **C** Partial least squares discrimination analysis (PLS-DA) score plot. **D** Permutation test for PLS-DA. **E** Variable importance (VIP) score of differentially abundant metabolite (VIP > 2, *P* < 0.05) content. **F** Metabolite counts associated with the differences between the LP and ANLP treatment groups. The color indicates the trend of metabolite levels in the LP treatment group compared with the ANLP treatment group (blue, upregulated; red, downregulated). CK, control group (no exogenous inoculant); AN, inoculation with *Aspergillus niger*; LP, inoculation with *Lactiplantibacillus plantarum*; ANLP, inoculation with *A. niger* and *L. plantarum*

**Fig. 5:**
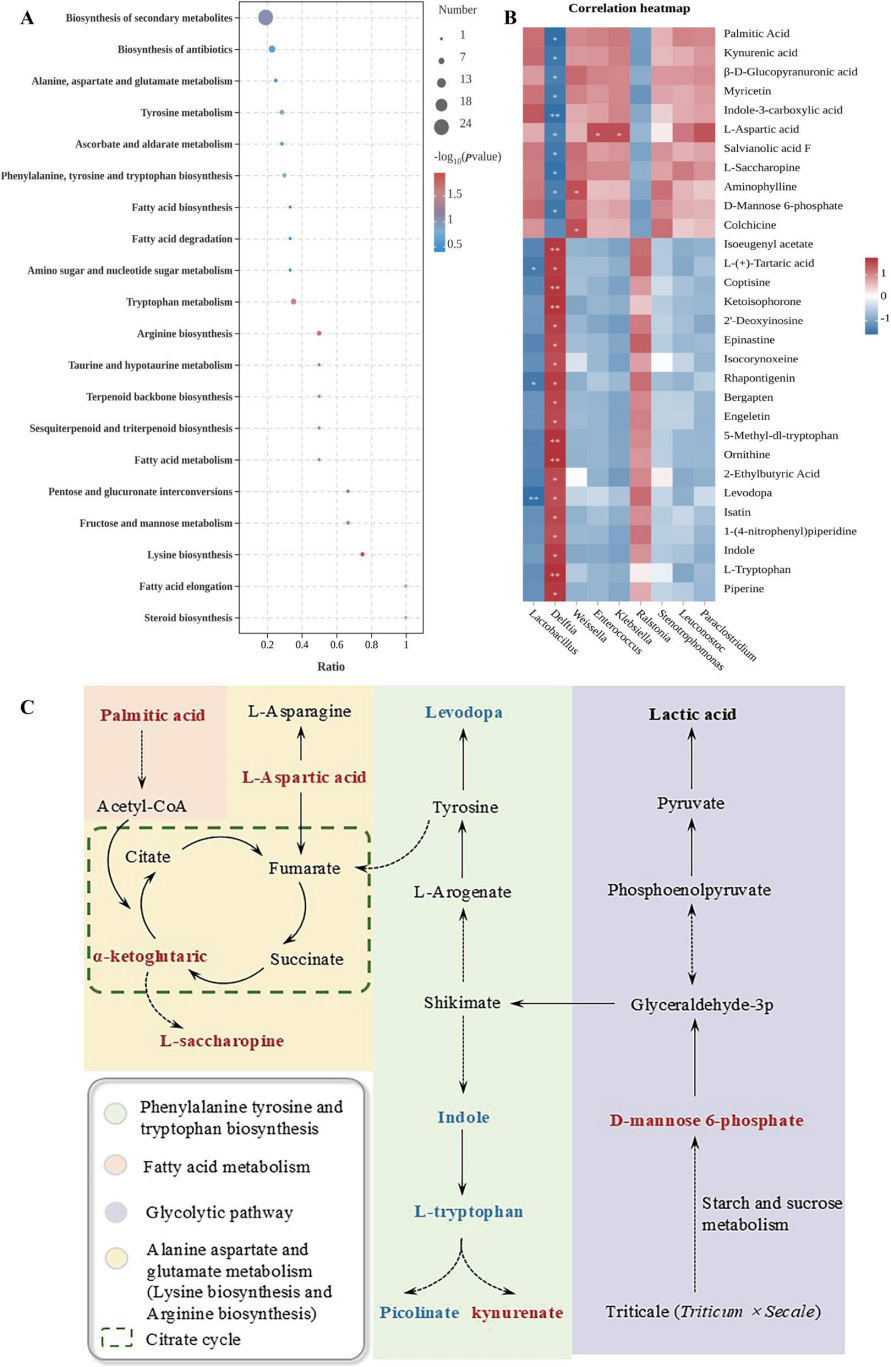
TThe KEGG enrichment, microbial correlation and metabolic pathway analysis of differential metabolites between the LP and ANLP treatment group.** A** Statistics for the KEGG enrichment analysis of differentially abundant metabolites. The bubble size represents the enrichment factor of the pathway, and the color represents the *P* value of the pathway. **B** Correlations between the 30 differentially abundant metabolites (VIP > 2 and *P* < 0.05) and bacterial genera based on the Spearman correlation coefficient (*P* < 0.05). **C** The potential metabolic pathways of the differentially abundant metabolites were reconstructed using the KEGG database. The phrases shown in red represent upregulated metabolites, while those shown in blue represent downregulated metabolites

By employing widely targeted metabolomics, we investigated the volatile metabolite profiles of triticale silage subjected to various treatments. As shown in Fig. S1, a comprehensive analysis revealed the presence of 1,279 volatile compounds, which were classified into 12 categories. Furthermore, PCoA and the corresponding PLS-DA score plots, which were derived from the metabolite profiles of triticale silage from various treatment groups, as shown in Fig. 4A and B, revealed significant metabolic variations in triticale silage treated with different additives. Based on the analyses of the chemical composition and fermentation quality, the fermentation performance of the LP and ANLP treatments was superior to that of the other treatments. Consequently, we employed metabolomics to investigate the effects of the LP and ANLP treatments on the metabolites, metabolic pathways, and metabolic networks in triticale silage. The differentially accumulated metabolites between the LP and ANLP treatment groups, which were analyzed using PLS-DA and permutation testing, are shown in Fig. 4C and D. Through an examination of the differentially abundant metabolites between the LP and ANLP treatment groups, a total of 212 metabolites were identified as significantly altered, with VIP values > 1 and *P* values < 0.05 (Table S1). A total of 213 differentially abundant metabolites were identified, and 130 were upregulated, whereas 83 were downregulated (Fig. 4E). Among the metabolites identified as differentially accumulated, lipids and lipid-like molecules (23), organic acids and organic acid derivatives (16), organoheterocyclic compounds (39), and phenylpropanoids and polyketides (35) were identified as the most active in triticale silage (Fig. 4E). The differentially abundant metabolites with VIP values > 2 and *P* values < 0.05 are presented in Fig. 4F. Compared with LP treatment, ANLP treatment resulted primarily in the downregulation of lipid and lipoprotein metabolites but also in the upregulation of organic acids and their derivative metabolites. An analysis of the data presented in Table S1 revealed that ornithine and citrulline levels were increased in ANLP-treated samples compared with LP-treated samples. Compared with the LP-treated samples, the ANLP-treated samples presented increased concentrations of vitamin C and L-tryptophan, whereas the tryptophol levels decreased. Compared with the levels observed following LP treatment, metabolites of the phenylpropanoid and polyketide classes, including 5-O-caffeoylshikimic acid, were downregulated following ANLP treatment. We conducted a KEGG pathway enrichment analysis of the identified metabolites to investigate the potential metabolic pathways associated with the differentially abundant metabolites. The results of this analysis revealed that the metabolic pathways associated with the differentially abundant metabolites between the LP and ANLP treatment groups included “tryptophan metabolism”, “arginine biosynthesis”, and “lysine biosynthesis” (*P* < 0.05; Fig. 5A).

### Correlation analysis of the microorganisms in terms of metabolites

In the LP and ANLP treatment groups, a total of nine bacterial genera, namely, *Lactiplantibacillus*, *Delftia*, *Weissella*, *Enterococcus*, *Klebsiella*, *Ralstonia*, *Stenotrophomonas*, *Leuconostoc*, and *Paraclostridium*, were identified and found to influence fermentation quality (Fig. 2E). Additionally, Fig. 5B shows that Spearman’s correlation analysis was performed to investigate the potential associations between these bacterial genera and 30 differentially abundant metabolites (VIP values > 2 and *P* values < 0.05), with the aim of elucidating the relationships between the microbiota and distinct differentially abundant metabolites. Furthermore, a metabolic network encompassing pivotal differentially abundant metabolites (VIP values > 2 and *P* values < 0.05) was constructed using the KEGG database (Fig. 5C). Importantly, *Lactiplantibacillus* was significantly negatively correlated with L-(+)-tartaric acid, rhapontigenin, and levodopa levels, whereas *Delftia* was significantly positively correlated with the levels of these compounds. Additionally, *Delftia* was significantly correlated with 29 differentially abundant metabolites. Kynurenic acid, a metabolite derived from tryptophan catabolism, was negatively correlated with *Delftia*. A negative correlation was observed between the abundances of aminophylline and *Delftia*. A negative correlation was observed between D-mannose 6-phosphate and *Delftia*. Isoeugenyl acetate, coptisine, ketoisophorone, epinastine, isocorynoxeine, rhapontigenin, bergapten, engeletin, 5-methyl-DL-tryptophan, 2-ethylbutyric acid, levodopa, isatin, 1-(4-nitrophenyl)piperidine, indole, and piperine were positively correlated with *Delftia*. L-(+)-Tartaric acid, ornithine, and L-tryptophan were positively correlated with *Delftia*.

### In vitro degradation rate of triticale silage samples after 60 days of ensiling

The effects of 60 days of fermentation treatment on the in vitro degradation rates of triticale silage samples are shown in Table 1. Compared with the other treatment groups, the ANLP treatment group presented significantly higher IVDMD and IVNDFD (*P* < 0.05). Compared with the CK treatment group, the LP and AN treatment groups had a significantly higher IVCPD, whereas the ANLP treatment group had a significantly higher IVCPD than both the LP and AN treatment groups (*P* < 0.05).

**Table 1:** Effects of different additives on in vitro degradation rate of triticale silage samples after 60 days of ensiling

Items	CK	AN	LP	ANLP	*P* value
In vitro dry matter degradation rate (IVDMD) , %	41.37 ± 3.29^b^	42.80 ± 1.75^b^	43.25 ± 1.50^b^	48.35 ± 1.89^a^	< 0.001
In vitro neutral detergent fiber degradation rate (IVNDFD), %	49.74 ± 3.74^b^	51.36 ± 1.99^b^	51.88 ± 1.70^b^	57.68 ± 2.15^a^	< 0.001
In vitro crude protein degradation rate (IVCPD), %	51.07 ± 1.49^c^	53.33 ± 0.70^b^	53.94 ± 1.47^b^	65.53 ± 2.73^a^	< 0.001

*CK* Control group (no exogenous inoculant), *AN* Inoculation with *Aspergillus niger*, *LP* Inoculation with *Lactiplantibacillus plantarum*, *ANLP* Inoculation with *A. niger* and *L. plantarum*. Different letters indicate significant differences between treatments (*P* < 0.05)

## Discussion

The results of this study demonstrate that the ANLP treatment group synergistically improved the nutrient composition, fermentation quality, microbial community, and in vitro degradation rate of triticale silage after 60 days of ensiling. Through an integrated analysis of chemical composition, microbial community, metabolomics, and correlations between microbes and metabolites, we focused on the interaction between the two co-inoculated strains and how this interaction influenced the fermentation of triticale silage. The DM content in the LP treatment group was significantly higher than that in the other treatment groups, likely because the added homofermentative *L. plantarum* inhibited the growth of competing microbes, thereby reducing nutrient consumption and mitigating DM loss [20]. The CP content observed in this study is consistent with that in previous reports (105 g/kg DM) for triticale silage [1]. The superior CP retention in the ANLP treatment group aligns with the findings of Zhai et al. [5], who reported that the addition of *A. niger* reduces CP loss in ratooning sorghum silage. During the ensiling process, numerous factors influence plant protein hydrolysis, and *A. niger* has been shown to modify the microbial distribution, subsequently altering the activity of enzymes involved in protein hydrolysis and reducing CP consumption [5]. Furthermore, inoculation with *L. plantarum* has been shown to inhibit the growth of proteolytic bacteria [20], which, in synergy with coinoculated *A. niger*, minimized CP losses and likely contributed to the highest CP content in the ANLP treatment group. The ANLP treatment group presented the lowest levels of NDF and ADF but the highest WSC content. These findings are consistent with those reported by Zhai et al. [5], who demonstrated that the incorporation of *A. niger* effectively decreases the NDF and ADF contents in ratooning sorghum silage. This effect is attributed to the secretion of various enzymes by *A. niger*, including cellulase, which catalyzes fiber degradation [6]. Furthermore, *L. plantarum* alters the microbial environment by lowering the pH and producing acids, thereby indirectly promoting fiber degradation [8]. Consequently, coinoculation with *L. plantarum* and *A. niger* increased the extent of NDF and ADF degradation, resulting in a corresponding increase in the WSC content.

The lower pH and higher LA content in the additive-treated groups suggest that the additives promoted the conversion of WSC to organic acids, leading to a subsequent decrease in pH, which is consistent with the observations reported by Li et al. [1] in their investigation of triticale silage. The decrease in pH is attributed primarily to the accumulation of organic acids [20], with the ANLP treatment resulting in the highest LA concentration. This outcome is attributed to the rapid consumption of oxygen in silage by *A. niger*, which accelerates the shift to an anaerobic environment [5]. The metabolic activity of *A. niger* not only promotes fiber degradation, thereby increasing the amounts of substrates available for LAB to synthesize LA, but also increases the internal temperature of the silage, creating optimal conditions for the rapid proliferation of LAB [21]. Consequently, coinoculation with *A. niger* and *L. plantarum* led to an increase in the LA content. The higher AA content in the ANLP and AN treatment groups is consistent with the findings of Liu et al. [21], who reported that corn stalks inoculated with *A. niger* presented the highest AA content. The elevated PA content in the AN treatment group is attributed to the inherent metabolic pathways of *A. niger*, which directly synthesize PA as a secondary metabolite. The presence of PA in *A. niger* metabolic extracts, as previously reported by Soni et al. [22], further corroborates its role in increasing PA accumulation during fermentation. The NH_3_-N content is a well-established indicator of protein degradation. The decrease in the NH_3_-N content in the LP and ANLP treatment groups can be attributed to the ability of *L. plantarum* to increase the concentrations of acids, particularly LA and AA, resulting in a rapid decrease in pH, which inhibits the activities of plant proteases and protein-degrading bacteria [1]. While *A. niger* can consume a significant portion of the available oxygen and thus suppress protein degradation by aerobic bacteria [21], its inhibitory effect appears to be limited. Therefore, the combined action of *A. niger* and LAB likely contributed to a more substantial reduction in CP degradation. These improvements of fermentation quality and nutrient composition in triticale silage were closely linked to the synergistic interactions between the bacterial and fungal communities within the silage.

The observation of reduced microbial abundance and diversity in the LP and ANLP treatment groups is consistent with the findings of Li et al. [1]. This outcome may be attributed to the inhibitory effect of the low-pH environment in the LP and ANLP treatment groups (Fig. 1F) on the growth of other bacterial species. The increased relative abundance of Firmicutes and decreased relative abundance of Proteobacteria in additive-treated groups are consistent with observations in triticale silage reported by Li et al. [1]. Researchers have demonstrated that spherical LAB, including *Lactococcus*, *Enterococcus*, and *Weissella*, predominantly exhibit robust growth during the initial phases of ensiling [23]. This result suggests that ensiling mediated by the CK treatment (natural fermentation) progressed more slowly and remained incomplete even after 60 days of fermentation. Therefore, additives are needed to enhance the fermentation process of triticale silage. The dominance of *Lactiplantibacillus* in both the AN and LP treatment groups aligns with the findings of Liu et al. [21], who identified *Lactiplantibacillus* as the primary genus involved in the ensiling of reed straw inoculated with *A. niger*. These findings also corroborate the findings of Zhang et al. [24], who reported that *Lactiplantibacillus* was dominant during the ensiling of alfalfa inoculated with *L. plantarum*. Although *Lactiplantibacillus* was dominant in both the AN and LP treatments, the AN treatment resulted in relatively high abundances of *Weissella* and *Enterococcus*. This result accounts for the inferior fermentation quality observed in the AN treatment group relative to that in the LP treatment group, suggesting incomplete fermentation in the AN treatment group. Nonetheless, the relative abundance of *Lactiplantibacillus* in the AN treatment group surpassed that in the CK treatment group, whereas the relative abundances of *Weissella* and *Enterococcus* decreased. These findings indicate that *A. niger* positively influences the ensiling quality of triticale and that the application of exogenous microbial additives can modify the bacterial community composition in triticale silage. However, the duration of ensiling must be extended when these additives are utilized to obtain the optimal results. The ANLP treatment group significantly increased the relative abundance of *Delftia*, a genus commonly found in soil and plants that can degrade phenolic compounds and aniline [25]. The enumeration of *Delftia* after the ANLP treatment group further corroborated this finding (Fig. 2H). Although *Delftia* has rarely been reported in silage, it has been associated with pH reduction and cellulase production in sainfoin [26] and hybrid *Pennisetum* silage [27]. Despite crops variations, the core mechanism may be that *Delftia* drives a decrease in pH, thereby potentially promoting the growth of LAB. Although using aniline as the sole carbon and nitrogen source for selective plating to enumerate *Delftia* is a reasonable approach, the lack of taxonomic confirmation of the counted colonies means that the medium cannot completely exclude other bacteria that degrade aniline, which constitutes a limitation of this study. To address this issue, our future research will incorporate 16S rRNA gene sequencing technology to identify representative colonies growing on the selective plates. This strategy will distinguish *Delftia* from nontarget aniline degrading bacteria and establish a correspondence between molecular identification and plate counts.

In terms of the fungal community, Ascomycota was predominant across all the treatment groups, which was consistent with the findings of Li et al. [28], who reported that Ascomycota was the predominant fungal phylum in triticale silage. The relative abundance of Ascomycota was higher in the additive-treated groups than in the CK treatment group. This increase could be attributed to the inoculation of *A. niger*, a member of Ascomycota, in the AN and ANLP treatment groups, whereas in the LP treatment group, the lower pH might have inhibited acid-sensitive fungi, thereby allowing acid-tolerant Ascomycota members to become dominant. At the genus level, *Cladosporium* and *Alternaria* were dominant in the CK treatment group. Both are aerobic fungi commonly found on fresh plant materials [29, 30]. Their persistence after 60 days of fermentation indicates that the transition to anaerobic conditions during natural fermentation was slow and incomplete. This prolonged aerobic phase allowed these obligate or facultative aerobic fungi to remain metabolically active for an extended period, continuously consuming WSC and degrading CP. Consequently, the delayed establishment of an anaerobic and acidic environment in the CK treatment group likely resulted in poor nutrient preservation, which accounts for the elevated pH and increased NH_3_‑N content observed in this treatment. In the LP treatment group, the fungal community became dominated by *Candida* and *Pichia*. The rapid pH decline caused by the inoculated *L. plantarum* effectively inhibited the growth of most acid-sensitive fungi, but its inhibitory effect on these acid-tolerant yeasts was limited [31]. Both *Candida* and *Pichia* can utilize residual sugars for fermentative metabolism, and some species are also capable of assimilating organic acids such as LA under certain conditions. Therefore, their dominance in the LP treatment group represents a potential risk. Although they did not cause outright spoilage during the 60 days of fermentation period, their presence may predispose the silage to aerobic deterioration upon silo opening, as yeasts are often the initiators of aerobic spoilage. In the AN and ANLP treatment groups, *Aspergillus* emerged as the dominant genus, confirming the successful colonization of the inoculated *A. niger* in triticale silage. *A. niger* is particularly well suited to the silage environment because it can maintain metabolic activity across a wide pH range (2.0 to 8.0) and can grow under the low-oxygen conditions that develop during the early stage of ensiling [32]. Its introduction into silage not only directly altered the fungal community composition but also indirectly reshaped the bacterial community structure through the provision of hydrolytic products. *A. niger* can secrete a diverse array of extracellular enzymes, including cellulases, hemicellulases, pectinases, and proteases. These enzymes likely initiated the partial degradation of structural polysaccharides, releasing WSC that served as substrates for LAB, thereby contributing to the accelerated pH decline observed in these treatments. In the ANLP treatment group, a marked increase in the relative abundance of *Aspergillus* coincided with the suppression of spoilage‑associated fungi, indicating a synergistic effect unattainable with either inoculant alone. This interpretation is supported by Liu et al. [33], who showed that inoculation with *A. niger* altered the fungal and bacterial communities during the fermentation of Baoning vinegar and that treatments with higher relative abundances of *Aspergillus* also resulted in higher relative abundances of *Lactiplantibacillus* and lower relative abundances of *Candida* and *Pichia*. This synergy likely arose from temporally coordinated reciprocal facilitation between the bacterial and fungal communities. During the early fermentation phase, the inoculated *L. plantarum* rapidly produced LA, quickly lowering the pH to approximately 4.0 to 4.5. This pH range was favorable for the growth and enzyme activity of *A. niger* while inhibiting many competing fungi and acid‑sensitive bacteria, thereby allowing *A. niger* to become dominant. As fermentation progressed, extracellular enzymes secreted by *A. niger*, such as cellulase, pectinase, and hemicellulase, likely degraded structural polysaccharides into fermentable sugars, continuously supplying substrates for *L. plantarum* and other acid‑tolerant microorganisms. This sustained sugar supply helped *L. plantarum* maintain active LA production, thereby stabilizing the acidic environment and preventing premature fermentation arrest. The bacterial–fungal synergy was also reflected in the bacterial community: the ANLP treatment group not only maintained a relatively high abundance of *Lactiplantibacillus* but also significantly increased the abundance of *Delftia*. This mutual facilitation may have been further strengthened by the enrichment of *Delftia* in the ANLP treatment group. The low‑pH environment generated by *L. plantarum* was possibly reinforced by the acidogenic metabolism of *Delftia* itself, creating a more stable acidic environment that favored the activity of the hydrolytic enzymes of *A. niger*, which typically exhibit optimal performance under acidic conditions. Simultaneously, the cellulase released by *Delftia* may have acted synergistically with the cellulase of *A. niger* to degrade fiber more efficiently, releasing additional sugars that could be utilized by both *L. plantarum* and *Delftia*. This multi‑enzyme cooperation on lignocellulose further explains the reduction in NDF and ADF contents observed in the ANLP treatment group. These findings suggest that *A. niger* and *L. plantarum* can establish synergistic interactions during fermentation, thereby allowing each to achieve dominance within the bacterial and fungal communities, respectively, and contributing to the observed improvements in fermentation quality.

The correlation between the bacterial community and the nutrient composition and fermentation quality was further supported in the ANLP treatment, where a higher relative abundance of *Delftia* coincided with elevated LA levels and a lower pH value. *Delftia* species are known to lower pH and secrete cellulase [29], which may jointly inhibit proteolytic bacteria, reduce NH_3_‑N accumulation, and degrade fiber to release WSC [20, 29]. Although a correlation between the abundance of *Delftia* and the improvement in the fermentation quality of triticale silage was observed in this study, whether *Delftia* directly contributed to these improvements remains to be validated through targeted isolation and reinoculation experiments in the future. The heatmap of the correlation analysis revealed that the fungal taxa could be clearly divided into distinct functional groups based on their metabolic roles. *Aspergillus* and *Rhizopus* exhibited fermentation-promoting characteristics. Their abundances were significantly positively correlated with the levels of nutritional and fermentation indicators such as CP, WSC, and LA and significantly negatively correlated with the NDF content, ADF content, and pH value. These findings suggest that these fungi may secrete cellulases and hemicellulases to release fermentable sugars from structural carbohydrates, thereby supplying substrates for LAB, promoting LA accumulation, and inhibiting CP degradation, which ultimately contributes positively to fermentation quality. In contrast, *Cladosporium*, *Alternaria*, and *Fusarium* displayed characteristics associated with fermentation spoilage. Their abundances were significantly positively correlated with the NDF, ADF, and NH_3_-N contents and with the pH and significantly negatively correlated with the CP, WSC, and LA contents. These findings indicate that these fungi may compete with LAB for fermentable sugars, resulting in insufficient LA production and an elevated pH while also causing NH_3_-N accumulation through protein degradation, which is detrimental to nutrient preservation in triticale silage. *Candida*, *Pichia*, and *Saccharomyces* were only weakly associated with the levels of CP and organic acid indicators and played a minor overall regulatory role in the fermentation system. The synergistic modulation of the microbial community by *A. niger* and *L. plantarum* likely induced alterations in the metabolite profile. Consequently, we employed metabolomics to investigate the effects of the LP and ANLP treatments on the metabolites, metabolic pathways, and metabolic networks in triticale silage.

The metabolites identified in silage fermentation play distinct roles and have nutritional significance. The significant metabolic variations observed among the treatment groups, as indicated by PCoA and PLS-DA, demonstrated clear segregation and differentiation among the four treatment groups, with the AN and ANLP treatment groups clustering closely together. The results obtained are analogous to those previously reported by Du et al. [34], where significant alterations in the metabolites of wheat straw silage were observed following the addition of *L. plantarum*. PLS-DA provided valuable insights into the relationships among the treatments (Fig. 4C), yielding one predictive component and two orthogonal components [34]. As depicted in Fig. 4D, the permutation test (R^2^Y = 0.92, Q^2^Y = 0.74) confirmed the sufficient effectiveness of the model [9]. Piperine, a bioactive alkaloid that exhibits antimicrobial activity, likely modulates the silage microbial community structure by suppressing pathogens while promoting the growth of LAB. Its presence increases aerobic stability and reduces proteolysis during ensiling. Kynurenine, a key tryptophan metabolite and critical intermediate in the kynurenine pathway, reflects microbial nitrogen metabolism and stress responses. Collectively, these metabolites may regulate fermentation quality by modulating the microbial community, with piperine and kynurenine reflecting metabolic shifts that help explain the effects of the differentially abundant metabolites observed between the LP and ANLP treatment groups.

Compared with LP treatment, ANLP treatment resulted primarily in the downregulation of lipid and lipoprotein metabolites, suggesting that the combined application of *A. niger* and *L. plantarum* facilitated the more efficient utilization of lipids in metabolic processes. *L. plantarum* converts fatty acids to energy via lipid metabolism, where fatty acids serve as the primary energy source and maintain the organism’s vital life activities [32]. Similarly, *A. niger* produces esterase, an enzyme crucial for fat metabolism that catalyzes esterification and hydrolysis reactions to modulate fatty acid activation and translocation [7]. The citric acid cycle, a central pathway in lipid metabolism, converts fatty acids and other intermediates into energy or indispensable biomolecules [9]. Notably, *A. niger*, a major citric acid producer, increases fatty acid consumption in triticale silage [31], partly through its biosynthesis of lipase, an enzyme capable of hydrolyzing the ester bonds within fats to yield glycerol, fatty acids, and phospholipid acids [31]. These metabolites function as energy sources and serve as precursors for phospholipids, sphingolipids, and related lipids [7], thereby contributing to the upregulation of specific lipid and lipid-mimetic metabolites. Organic acids and their derivatives, which primarily include amino acids, amides, peptides, and their derivatives, play pivotal physiological roles in biological systems, influencing processes such as protein synthesis, hormone regulation, and cell signaling [32]. Compared with ANLP treatment, LP treatment resulted in the upregulation of organic acids and their derivative metabolites. These findings align with previous observations of wheat straw silage and Italian ryegrass silage, where inoculation with *L. plantarum* alone has been shown to increase the levels of metabolites associated with organic acids and their derivatives [34, 35]. The ability of LAB to facilitate the depolymerization of complex compounds to simpler compounds has been proposed as an explanation for these results [4]. In contrast, due to the presence of *A. niger*, ANLP treatment has enriched the enzyme types, leading to increased utilization of certain organic acids and their derivatives [31], and thereby causing their downregulation. Concurrently, these more intricate biochemical processes have also contributed to increases in the levels of other organic acids and their derivatives. An analysis of the data presented in Table S1 revealed that ornithine and citrulline levels were increased in ANLP-treated samples compared with LP-treated samples. Both ornithine and citrulline are integral intermediates within the urea cycle, a pivotal biochemical pathway responsible for converting the toxic compound ammonia to the nontoxic compound urea [32]. The observed increase in ornithine and citrulline levels is consistent with the lowest NH_3_-N levels recorded in the ANLP treatment group, suggesting that the microorganisms enriched in the ANLP treatment may have efficiently utilized the urea cycle to consume NH_3_-N, consequently increasing the concentrations of ornithine and citrulline. Organoheterocyclic compounds, including pyrazoles, imidazoles, isoindoles, indazoles, indole-3-carboxylic acids and their respective derivatives, display a spectrum of biological activities within living organisms [36]. Similar to the findings of this study, organoheterocyclic compounds are among the active metabolites detected in whole-plant corn silage [18]. This observation is attributed to protein hydrolysis being a major biochemical process during anaerobic fermentation, resulting in the production of many short peptides and amino acids [1]. Analogously, the proteolytic process induced by enzymes and microbial physiology and biochemistry also leads to changes in the nitrogen-containing compounds in silage [20]. Previous studies have demonstrated that organoheterocyclic compounds are ubiquitous in nature and hold paramount importance for life, playing a fundamental role in the metabolic processes of all living cells [32]. Some of them exhibit antibacterial and anti-inflammatory properties, enabling them to modulate the physiological and biochemical activities of microorganisms by inhibiting the proliferation of pathogenic bacteria while promoting the survival and multiplication of beneficial microbial species [37]. Compared with the LP-treated samples, the ANLP-treated samples exhibited increased concentrations of vitamin C and L-tryptophan, whereas the tryptophol levels decreased. Vitamin C has potent antioxidant properties, assisting LAB in mitigating cellular oxidative stress. This antioxidant capability is crucial for maintaining cellular homeostasis and functionality because it effectively scavenges free radicals and safeguards vital intracellular molecules from oxidative insults [38]. Moreover, previous research has established that the incorporation of L-tryptophan markedly increases the levels of WSC and LA in perennial ryegrass silage while concurrently reducing the abundance of *Clostridium* species [39]. Tryptophol, a metabolite generated by LAB, functions as a signaling molecule capable of modulating fungal morphogenesis and inducing apoptosis [20]. During ANLP treatment, inoculation with *A. niger*, coupled with the upregulation of other antioxidant metabolites, led to a decrease in tryptophol abundance. Phenylpropanoids and polyketides constitute a class of metabolites that exhibit diverse biological activities, including antibacterial, anti-inflammatory, and antioxidant activities [40]. The phenylpropanoid biosynthesis pathway predominantly involves the shikimate pathway, which subsequently leads to the formation of aromatic amino acids, notably phenylalanine [37]. Polyketides, on the other hand, represent a diverse array of secondary metabolites synthesized by bacteria, fungi, plants, and animals, encompassing tetracycline and macrolide antibiotics, as well as numerous other natural compounds [41]. Research suggests that phenylpropanoids and polyketides interact with beneficial soil microorganisms, promoting the formation of secondary cell walls and the production of lignin, whereas inoculation with *A. niger* increases the secretion of lignin-degrading enzymes, leading to the downregulation of phenylpropanoid and polyketide production [31].

The metabolic pathways associated with the differentially abundant metabolites between the LP and ANLP treatment groups included “tryptophan metabolism”, “arginine biosynthesis”, and “lysine biosynthesis” (*P* < 0.05; Fig. 4E). These findings are consistent with prior observations of the enrichment of “tryptophan biosynthesis” in Italian ryegrass silage [42]. Tryptophan metabolism regulates protein biosynthesis and generates bioactive compounds that influence inflammation, immunity, and neuronal activity while also increasing the antimicrobial capacity of LAB via protein posttranslational modifications [39]. Concurrently, arginine biosynthesis drives the urea cycle, converting toxic ammonia into ornithine, thereby minimizing protein degradation and conserving the CP content in triticale silage [43]. Lysine serves as a multifaceted and indispensable component within biological organisms and plays crucial roles in protein biosynthesis, acid‒base homeostasis maintenance, and growth and developmental processes [44]. Lysine is categorized as an essential amino acid, meaning that it cannot be synthesized de novo by animals [45]. Consequently, the augmentation of the lysine synthesis pathway in ANLP-treated triticale silage suggests a superior nutritional benefit for animals consuming this silage. To investigate the relationship between the altered microbial community and the metabolites, we performed a correlation analysis between the dominant microbial genera and the differentially abundant metabolites.

The findings revealed associations between five bacterial genera and a panel of 30 differentially abundant metabolites. *Lactiplantibacillus* was negatively correlated with L-(+)-tartaric acid, rhapontigenin, and levodopa levels, whereas *Delftia* was positively correlated with the levels of these same compounds, suggesting that *Delftia* improves triticale silage quality through distinct metabolic pathways. L-(+)-tartaric acid inhibits LAB growth, which explains its negative correlation with *Lactiplantibacillus* abundance [30]. Rhapontigenin and levodopa, which have diverse bioactivities, also inhibit *Lactiplantibacillus* growth [46, 47]. *Delftia* was negatively correlated with palmitic acid and L-aspartic acid levels because it catabolizes palmitic acid to acetyl-CoA and L-aspartic acid to fumarate for entry into the TCA cycle [48, 49]. The negative correlations with kynurenic acid and β-D-glucopyranuronic acid levels imply that *Delftia* may suppress tryptophan decomposition and utilize alternative carbohydrate pathways to conserve nutrients [49–51]. Myricetin, indole-3-carboxylic acid, and salvianolic acid F inhibit the growth of *Delftia*, which explains their negative correlations [52–54]. The negative correlation with L-saccharopine levels indicates that *Delftia* may accelerate its conversion to lysine, increasing its amino acid content [55]. A negative correlation with aminophylline levels suggests that *Delftia* reduces the production of this gastrointestinal irritant, mitigating digestive damage [56]. The negative correlation with the level of D-mannose 6-phosphate, which feeds glycolysis to produce LA, suggests that *Delftia* accelerates glycolysis, which is consistent with the higher LA content in ANLP-treated plants harboring abundant *Delftia* [9]. *Delftia* was positively correlated with numerous bioactive metabolites (e.g., isoeugenyl acetate, coptisine, ketoisophorone, epinastine, isocorynoxeine, rhapontigenin, bergapten, engeletin, 5-methyl-DL-tryptophan, 2-ethylbutyric acid, levodopa, isatin, 1-(4-nitrophenyl)piperidine, indole, and piperine) that exhibit antibacterial, antiviral, anticancer, anti-inflammatory, and antioxidant activities, suggesting that *Delftia* promotes the production of these compounds to suppress the growth of harmful bacteria [57–62]. The L-(+)-tartaric acid level was also positively correlated with the abundance of *Delftia*, which may secrete this metabolite to decrease the pH of triticale silage [45]. *Delftia* abundance was positively correlated with ornithine levels, likely enhancing the urea cycle to reduce NH_3_-N levels [32]. The positive correlation with L-tryptophan levels could mean that *Delftia* is able to synthesize this metabolite, thereby potentially improving silage quality [39]. These correlation data between the microbial community and the metabolome indicated that the enrichment of *Delftia* and the dominance of *Lactiplantibacillus* in the ANLP treatment group jointly drove a coordinated metabolic shift toward enhanced nitrogen retention, fiber degradation, and the production of antimicrobial compounds.

A metabolic network encompassing pivotal differentially abundant metabolites was constructed using the KEGG database (Fig. 5C). *A. niger* may secrete extracellular enzymes such as amylases to degrade complex carbohydrates into simple sugars, which provide substrates for glycolysis. *L. plantarum* could then efficiently ferment these sugars via glycolysis, converting glucose to LA and thereby potentially lowering the pH of the silage to preserve its quality. *A. niger* might produce intermediates in the phosphoketolase pathway as alternative fermentation products such as ethanol and AA, whereas *L. plantarum* would modulate flux to balance end-product profiles. Thus, *A. niger* plausibly primes carbohydrate substrates and *L. plantarum* drives glycolytic fermentation, together increasing overall carbohydrate metabolism and silage efficiency. In ANLP-treated triticale silage, starch and various sugars are likely metabolized predominantly through glycolysis and the phosphoketolase pathway, which promotes the consumption of D-mannose 6-phosphate and ultimately increases LA production. Collectively, these improvements in fermentation quality, nutrient composition, microbial community structure, and metabolomic profiles led to an enhanced in vitro degradation rate of triticale silage.

As shown in Table 1, compared with the other treatment groups, the ANLP-treated group exhibited significantly higher IVDMD and IVNDFD. These results are consistent with those of the previous analysis of the chemical composition, in which the ANLP group presented the lowest NDF and ADF contents and the highest WSC content. The effective degradation of fiber structures may have been a direct consequence of the synergistic cellulolytic activities of *A. niger* and the enriched *Delftia*, while the increased WSC content provided more readily available substrates for microbial fermentation, further promoting DM degradation. Regarding the IVCPD, the ANLP group also exhibited the best performance, significantly surpassing both the single-inoculant treatment groups and the CK treatment group. Although it had the highest CP content, its IVCPD was also the highest, suggesting that this treatment may have better preserved protein and potentially enhanced protein digestibility. This improvement may be attributed to the synergistic action of microorganisms and enzymes: *A. niger* secreted proteases that promoted CP hydrolysis, while *L. plantarum* suppressed CP-degrading bacteria through rapid acidification. Moreover, the enriched relative abundance of *Delftia*, *Lactiplantibacillus*, and *Aspergillus* in the remodeled microbial community further upregulated the urea cycle and amino acid biosynthesis pathways, as indicated by metabolomics analysis. The accumulation of amino acids such as ornithine, citrulline, and lysine not only enhanced the nutritional value of silage but also provided readily fermentable nitrogen substrates, thereby indirectly promoting in vitro protein degradation. Thus, the enhanced in vitro digestibility likely represents the final outcome of the microbial community shifts and the consequent alterations in the metabolite profile driven by the coinoculation of *A. niger* and *L. plantarum*.

In summary, the combined application of *A. niger* and *L. plantarum* appears to have synergistically improved triticale silage quality through a multi-layered mechanism: *L. plantarum* rapidly lowered the pH value, creating a favorable environment for *A. niger* growth and enzyme activity; *A. niger* secreted hydrolytic enzymes that degraded lignocellulose and provided fermentable sugars for *L. plantarum*; their mutualistic interaction reshaped the microbial community by promoting beneficial bacteria such as *Delftia* and suppressing spoilage microorganisms; this remodeled microbial community further regulated key metabolic pathways, including nitrogen metabolism and amino acid biosynthesis; and collectively, these changes may have led to improved fermentation quality, enhanced nutritional value, and a higher in vitro degradation rate. These proposed mechanisms require further experimental validation. Future metagenomic studies will investigate the regulatory mechanisms of key metabolites to provide more robust evidence. The functional roles of *Delftia* will be verified through mechanistic experiments, including monoculture and coculture studies. Subsequent work will evaluate the practicality of the mixed microbial inoculant by comparing cultivation processes, costs, improvements in forage quality, and increases in sales revenue and will discuss influencing factors to guide industrial application.

## Conclusion

In summary, the results of this study demonstrated that the synergistic effects of *A. niger* and *L. plantarum* (ANLP treatment) significantly increased triticale silage quality by reshaping microbial communities and metabolic pathways. The synergistic effects of the bacterium (*L. plantarum*) and fungus (*A. niger*) promoted the dominance of *Delftia*, which played a dual role in optimizing fermentation: *Delftia* increased organic acid biosynthesis through aromatic amino acid metabolic pathways while simultaneously promoting the production of antimicrobial and antioxidant metabolites via fatty acid metabolism, thereby supplying energy substrates to optimize silage fermentation. These mechanisms collectively increase the nutrient retention, fermentation quality and in vitro degradation rate of triticale silage by effectively preserving CP and WSC, optimizing acidic conditions through a reduction in pH, and increasing LA production. Furthermore, future studies should prioritize the isolation of *Delftia* strains from the silage microenvironment for monoculture or coculture experiments to validate the mechanistic role of *Delftia* in silage enhancement.

## Supplementary Information

Additional file 1: Table. S1 The differentially abundant metabolites between the LP and ANLP treatment groups.

Additional file 2: Fig. S1 Proportions of different types of metabolites of triticale silage samples after 60 days of ensiling.

## Data Availability

The datasets used and/or analyzed during the current study are available from the corresponding author upon reasonable request. The NCBI database already includes the raw sequencing data under accession number PRJNA1328668 and PRJNA1457705. Our metabolomics data were uploaded to the NGDC OMIX repository, with the project number PRJCA046474.

## Abbreviations

AA: Acetic acid
ADF: Acid detergent fiber
AN: Inoculation with *Aspergillus niger*
ANLP: Inoculation with *A. niger* and *L. plantarum*
ANOVA: Analysis of variance
BA: Butyric acid
CK: Control group (no exogenous inoculant)
CP: Crude protein
DM: Dry matter
FM: Fresh weight
GRAS: Generally recognized as safe
HPLC: High-performance liquid chromatography
IVCPD: In vitro crude protein degradation rate
IVDMD: In vitro dry matter degradation rate
IVNDFD: In vitro neutral detergent fiber degradation rate
LA: Lactic acid
LAB: Lactic acid bacteria
LC‒MS: Liquid chromatography‒mass spectrometry
LP: Inoculation with *Lactiplantibacillus plantarum*
MRS: Rogosa and Sharpe
NDF: Neutral detergent fiber
NH_3_-N: Ammonia nitrogen
OTU: Operational classification unit
PA: Propionic acid
PCoA: Principal coordinate analysis
PDA: Potato dextrose agar
PLS-DA: Partial least squares discriminant analysis
QC: Quality control
RDA: Redundancy analysis
TN: Total nitrogen
WSC: Water-soluble carbohydrate
