# Supplementary figures and images for: Synergistic bacterial‒fungal interactions modulate the fermentation quality and in vitro degradation rate of triticale silage
Source: J Anim Sci Biotechnol. 2026 Aug 3;17:152. doi: 10.1186/s40104-026-01478-9 (PMC13430836; doi:10.1186/s40104-026-01478-9)

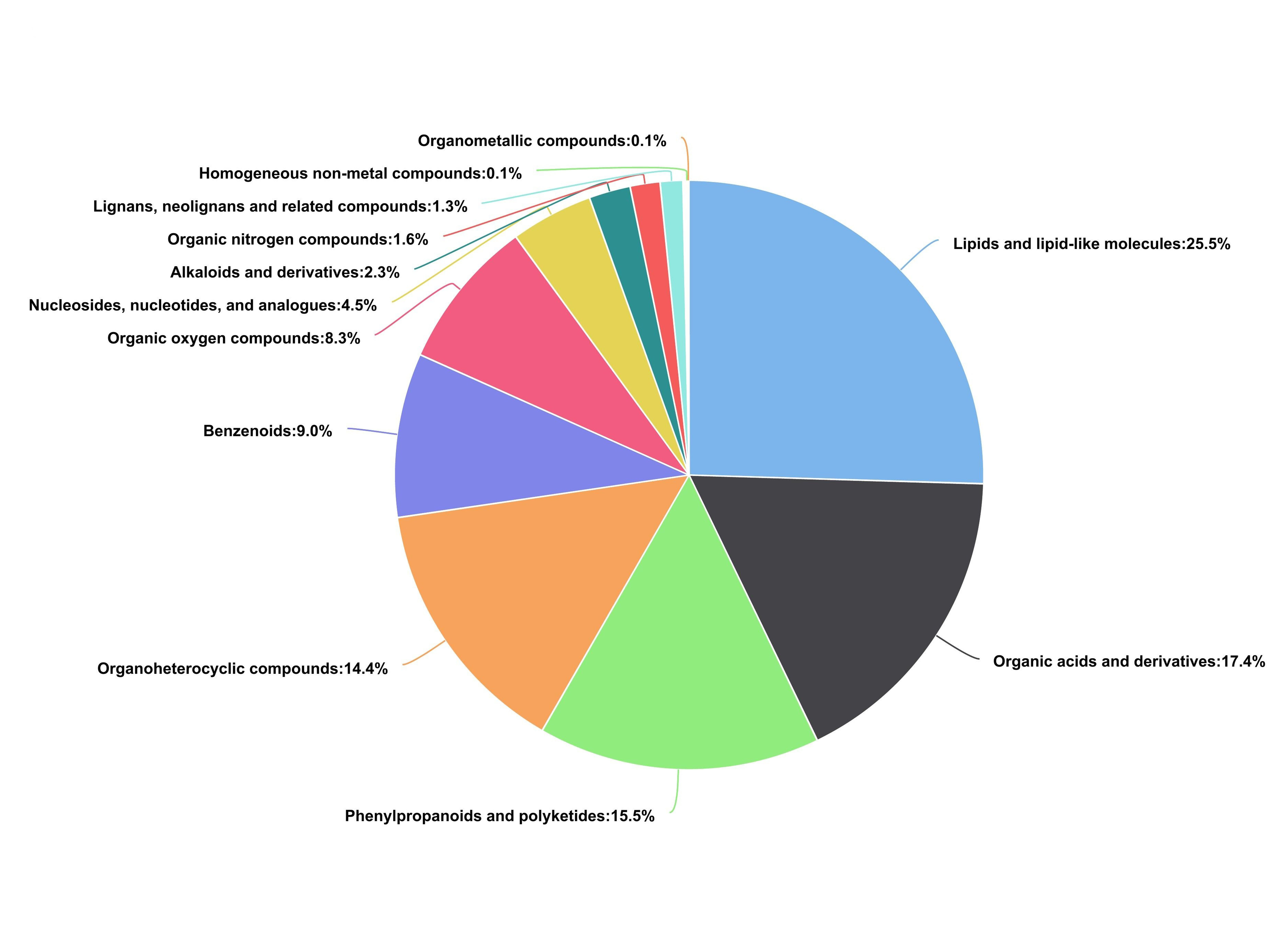

Supplement: Supplementary file 2 — Additional file 2: Fig. S1 Proportions of different types of metabolites of triticale silage samples after 60 days of ensiling. [file 40104_2026_1478_MOESM2_ESM.jpg]
